# A step towards microlitter risk assessment: modelling microlitter storage potential of the UK seabed

**DOI:** 10.1098/rsta.2024.0428

**Published:** 2025-10-23

**Authors:** Adil Bakir, Adam Porter, Ceri Lewis, Jon Barry, Robert Brookes, William Procter, Briony Silburn, Alexandra Rachael McGoran, Clement Garcia, Claire Mason, Stefan Bolam, David Stephen Clare, Keith Cooper, Anna Downie, Jim Ellis, Daniel Wood, Claire Phillips, Tamara S. Galloway

**Affiliations:** ^1^ Centre for Environment, Fisheries and Aquaculture Science (Cefas), Pakefield Road, Lowestoft, Suffolk NR33 0HT, UK; ^2^ College of Life and Environmental Sciences, Biosciences, Geoffrey Pope Building, University of Exeter, Stocker Road, Exeter EX4 4QD, UK

**Keywords:** seafloor, risk exposure, microplastics, sources, receptors

## Abstract

Seafloor sediments have been defined as sinks for microplastics in the marine environment and could therefore represent suitable matrices for their long-term monitoring. Previous studies indicated the widespread distribution of microlitter in seafloor sediments for the UK. In the present study, observations from 2017 to 2021 were used to produce a microlitter distribution model (unitless), derived from physical properties of the seabed that are known to drive the storage capacity of microlitter. The predicted distribution model was converted into a geospatial data layer and plotted against additional open access data layers for likely sources of marine litter (e.g. marine structures) as well as data layers for more sensitive features around the UK (e.g. marine protected areas (MPAs)). Visualization of the accumulation zones for microlitter against the different layers allowed the identification of areas potentially at risk from an increased addition of microlitter from various sources (e.g. dredge disposal sites). Identification of potential risks and prioritization for different zones of action would help the development of national and regional monitoring strategies while reducing costs of multi-compartment, larger scale monitoring programmes. Additional observations and targeted monitoring data are needed to link potential sources of accumulations for microplastics with a higher level of certainty.

This article is part of the Theo Murphy meeting issue ‘Sedimentology of plastics: state of the art and future directions’.

## 1. Introduction

Marine litter is defined as any solid material which has been deliberately discarded or unintentionally lost on beaches, on shores or at sea. The definition covers materials transported into the marine environment from land by rivers, draining or sewage systems or winds. It also includes any persistent, manufactured or processed solid material [1]. Marine litter pollution is a growing problem and monitoring programmes are an important tool to evaluate both the trends and the efficiency of litter reduction measures [2–4]. An important proportion of marine litter is composed of smaller sized plastic items below 5 mm in size, referred to as microplastics [1]. Microplastics can originate from both land and sea-based sources and from primary (e.g. pre-production plastic pellets) or secondary sources (i.e. resulting of the degradation of larger debris). Microplastics can also be referred to as ‘non-conventional’ contaminants due to their diversity in relation of size, morphologies and polymer type. The diverse range of microplastic types in the environment is increasing the difficulty of their detection and reporting in environmental samples [5]. Microplastics are ubiquitous in the marine environment and have been reported on the seabed [6–11], biota [12–17] and surface waters and water column [9,18–20]. Microplastics are recognized as a potential global threat to marine ecosystems and human health [21,22].

Microplastic can be ingested by a large range of marine organisms representing various trophic levels including seabirds, marine mammals, fish and invertebrates [23]. Detrimental physical effects of microplastics have been reported following ingestion [24]. There is evidence that microplastics can act as carriers for harmful sorbed co-contaminants (e.g. hydrophobic organic compounds, additives, pathogens) with the potential for transfer to biota following ingestion [25–30]. Microplastics are known to be widespread in the marine environment and have been found in every marine niche investigated, from coastal zones to the open ocean and the deep sea [6,11,13,31,32]. However, the effects of microplastics on seafloor ecosystem function remain unclear.

Seafloor sediments have also been suggested as a likely sink for microplastics [6,33]. Understanding microplastics abundance, fate and distribution on and within the seabed is important, as this would allow the development of robust monitoring programmes to evaluate both the trends and the efficiency of reduction measures. High spatial resolution monitoring would allow high-resolution maps of microplastic concentrations around the UK to be generated, affording the potential to derive exposure maps by overlaying hotspots and known sensitive or protected environments (e.g. fish breeding grounds, marine protected areas (MPAs)). Identification of such vulnerable areas would provide the basis for relevant policy action. Temporal comparisons of baseline concentrations would also characterize changes in concentrations following the implementation of new regulatory actions or best practices. Robust marine microplastic concentration data would also help guide dose–response ecotoxicological laboratory experiments and human health studies, allowing them to be conducted using environmentally realistic concentrations [34].

Monitoring data contributes towards the understanding of the properties and quantities of marine litter in coastal and marine sediments which aligns with the UK Government Marine Strategy and future requirements of the Convention for the Protection of the Marine Environment of the North-East Atlantic (the OSPAR Convention). At the OSPAR level, a proposed new common indicator for microplastics in seafloor sediments is also being developed. Robust monitoring strategies will also align with the objectives of the UN Sustainable Development Goals (Goal 14: Life below water) [35]. As the final sink for microplastics in the marine environment, the seafloor and associated sediments have been recommended as components within a marine litter monitoring programme [11,31,36]. Previous work suggested the widespread occurrence and distribution of microplastics in seafloor sediment for the UK (England and Wales). Kukkola *et al*. [37] and Bakir *et al*. [11] applied a fast-screening method (i.e. Nile Red) for the large-scale mapping of microplastics for seafloor sediments for the UK (England and Wales). Nile Red was developed as a low-cost and fast approach for the detection and quantification of microplastics in environmental samples [38]. Nile Red has been used for the large-scale mapping of microplastics from sediment, indicating its suitability in a monitoring context [37,39–41]. Nile Red has also been applied to the detection and quantification of microplastics in biota [42–45] and water [39,40]. Kukkola *et al*. [37] reported an average abundance of microplastics in the top 10 cm of 1050–2700 particles kg^−1^ d.w. sediment for particles (in the size range approx. 10 to 5000 μm) in three contrasting areas of the UK continental shelf (northwest of Jones Bank, the Canyons in the Celtic Sea and Dogger Bank in the North Sea). A multiple-year study also indicated the widespread distribution of microplastics in UK seafloor sediment with an abundance ranging from 0 to 6933 particles kg^−1^ d.w. sediment (in the size range approx. 20 to 5000 μm) with a mean value of 3057 ± 2299 particles kg^−1^ d.w. sediment for the years 2020 and 2021 [11]. Microplastics have been shown to present a threat to marine ecosystems and biodiversity globally [46]. However, there is a crucial lack of risk assessment of microplastics in global hotspots of marine biodiversity [47]. While international efforts are being made to develop global policy tools to reduce plastic pollution, scientific evidence is needed to support those efforts and to prioritize zones of action [48].

At a regional level the OSPAR Commission approved its first common indicator for microlitter (including microplastics) in seafloor sediment for the OSPAR Maritime Area [1]. The OSPAR Microplastic Expert group (or MPEG) recently published some monitoring guidelines [49] harmonized with other regional or international guidelines [50]. At a more global level, understanding occurrence, abundance and composition of microlitter on to the seabed would contribute towards reducing the effect from plastics (SDG indicator 14.1.1b under Gol 14).

The main aim of this study was to propose a geospatial risk-exposure map of microplastics on the seabed, to guide scientists and policy makers on priority areas for appropriate management action. The objectives were to (i) create a model to estimate distribution of microplastics on the seabed for the UK (England and Wales) using data from 2017 to 2021, (ii) identify accumulation zones or ‘hotspots’ for microplastics on the seafloor and (iii) propose a risk-exposure map for microplastics on the seabed for the UK with the identification of predicted zones at risk due to elevated abundance of microlitter.

## 2. Material and methods

### (a) Visualization of available data

The present study was based on data collected between 2017 and 2021. The 2017 microplastics sediment data (top 5 cm) were acquired by Kukkola *et al.* [37] for the northwest of Jones Bank and the Canyons in the Celtic Sea, and Dogger Bank in the North Sea. Data from 2018–2021 were collected as part of the Clean Seas Evidence Monitoring Programme (CSEMP) survey [11]. Additional information on CSEMP sediment stations is shown in electronic supplementary material, fig. S1 and table S1. Earlier CSEMP data, from 2013 to 2017, from Bakir *et al*. [11] were not included in this study as best practices for sample collection and quality control (e.g. contamination control measures) were applied only from 2018 onwards.

### (b) Prediction model

There are currently insufficient spatial microplastic data to allow a robust predictive spatial map of microplastic concentrations within seabed sediments for the UK or elsewhere. Instead, we used the available data to derive information on the physical makeup of the seabed that makes it efficient at storing the microplastic or not (regardless of whether there are some to be stored in the first place). As such, the result of the model is expected to be a ‘unitless’ microplastic storage potential of the seabed.

The boosting machine learning approach [51,52] was used to find the best subset of predictor variables of microplastic levels (carried out with the R package *gbm* [53]). The advantage of this approach is that it can accommodate nonlinear relationships of the predictor variables with the dependent variable and interactions between the predictor variables. The boosting method is a tree-based machine learning approach that has some similarities to the, perhaps more familiar, random forest method. Our experience is that boosting performs marginally better than random forest, and this is backed-up by Chollet & Allaire [54] who state that ‘boosting mostly out-performs random forest’ [54]. Our boosting model assumes that the number of microplastic particles is a function of some combination of 10 predictor variables (electronic supplementary material, table S3) suspected to affect the fate and distribution of microlitter on the seabed (e.g. sediment grain size, curvature of the seafloor terrain, salinity, bottom currents, etc. (denoted by $X_{1},X_{2},\ldots ,X_{10}$ in equation (2.1) below)).


(2.1) $$MP=f\left(X_{1},\;X_{2},\ldots ,X_{10}\right).$$

To find the best subset of explanatory variables for prediction of the number of microplastic particles, we need a measure of model performance. One common approach, the validation set method, is to use a form of cross-validation where models are fitted on approximately two-thirds of the cases (the training dataset) and then their prediction performance is evaluated on the cases not used to fit the model (the testing dataset). We did not use this method here because we had a relatively small number of complete observations (56) and we were concerned that our testing data set models would not have sufficient cases to fit good models. Instead, we used another form of cross-validation known as ‘leave one out’ (LOO) cross validation. We can think of this as having a testing dataset of size *N*-1, where only one case is removed (*N* is the number of cases). As before, the performance of the model is evaluated by comparing the actual and predicted values of the left-out case. This is repeated for all *N* cases, with a different one being left out each time. The LOO approach has an additional advantage over the validation set approach in that there is no variation due to the random choice of testing and training data sets. Its main disadvantage is that there are fewer cases to use for model evaluation.

Formally, we can define the LOO mean absolute error as


(2.2) $$LOOMAE=\;\frac{1}{N}\sum_{j=1}^{N}|MP_{j}-\hat{MP_{j}}|.$$

**Figure 1.**
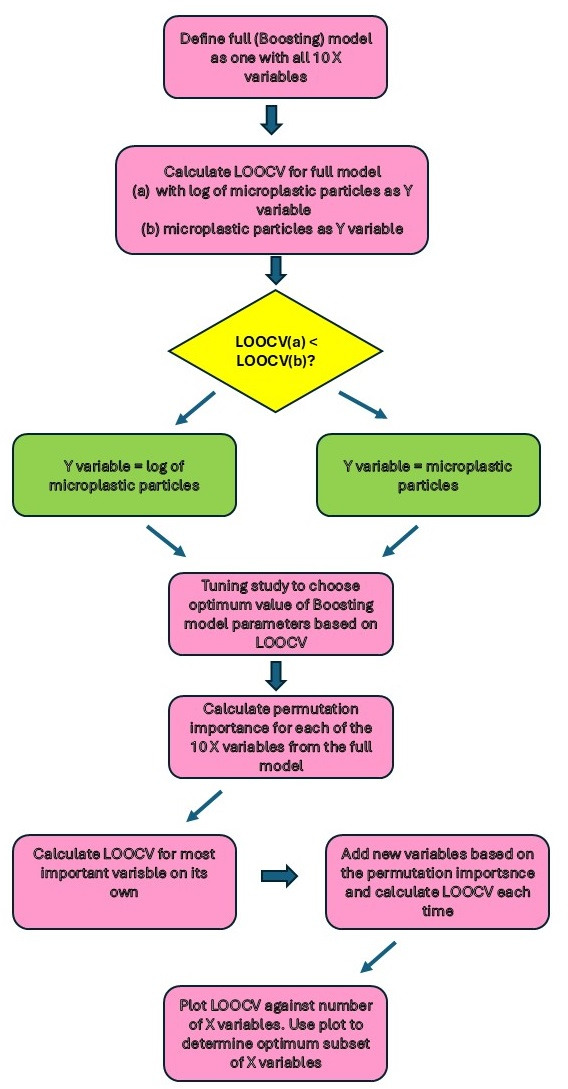
Flow chart to illustrate the way that the boosting models are used to find the best subset of the 10 predictor variables (X) to predict number of microplastic particles (Y).

**Figure 2.**
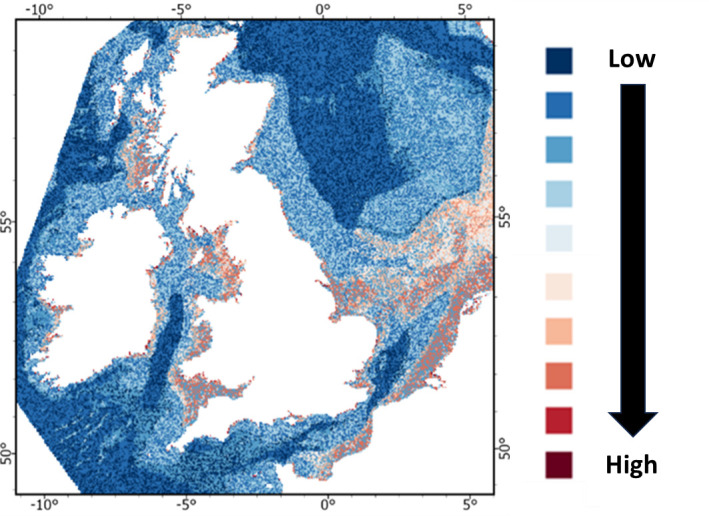
Abundance of microplastics on the seafloor for the UK EEZ based on its microplastic storage potential as predicted by our model. Abundance is unitless and therefore relative.

Absolute error was used instead of squared error so as not to give undue weight to any isolated points with bad predictions. Roberts *et al.* [55] noted that cross-validation methods can potentially lead to overfitting (and therefore an underestimate of the prediction error) if the dependent variable observations are spatially correlated [54]. Even though there is potential for spatial auto-correlation due to the close proximity of some of our points (see figure 2), our semi-variogram plots of model residuals did not show any obvious spatial autocorrelation. The boosting algorithm is stochastic in nature and so two successive runs of identical models do not yield the same results, especially with small datasets such as ours. We removed most of this variation by evaluating the mean LOOMAE from 100 runs of the model. We also ran simulation experiments using LOOMAE to determine the optimum value of the boosting parameters for use in our prediction models. For these simulations, we used all 10 predictor variables. These experiments suggested that we should use the untransformed values of microplastics (as opposed to a log transformation). They also concluded that the best parameters for the R *gbm* function were *ntrees* = 400, *depth* = 1 and *minobs* = 10. We used these parameter values for all further boosting models fitted in our study. The procedure for model selection was to first calculate the permutation importance measure for each variable [56] using the full model containing all 10 predictor variables. As with LOOMAE, our permutation importance values are based on 100 replications of the boosting models. While we prefer LOOMAE for model selection, permutation importance is an easy to calculate measure which we used to determine the order in which variables were added to the boosting model for calculation of LOOMAE. We then calculated LOOMAE for a succession of models, starting with the most important predictor variable as determined by the permutation importance measure and then successively adding more predictor variables until all 10 were included in the model. A plot of LOOMAE against number of predictor variables was used to show the optimum number of predictor variables to use for our best model. The boosting modelling procedure described above is illustrated by the flow diagram in figure 1. The best model for predicting microplastics was used to calculate the microplastic prediction map. The corresponding layer for microplastics is shown in figure 2.

### (c) Development risk exposure map

A number of geospatial data layers were used to understand the spatial relationship between our modelled sediment microplastics distribution and those of several anthropogenic pressures and ecologically important areas (i.e. those of designated conservation status and those of biological concern). The layers were: (i) UK dredged material disposal sites, (ii) aggregates site agreements, (iii) wind site agreements, (iv) marine protected areas (MPAs), (v) fish nursery and spawning grounds and (vi) key benthic invertebrate species that are expected to be sensitive to, or to amplify, the effects of microlitter. These layers, and how they relate to the present study, are listed in table 1, while further details on their sources are provided in electronic supplementary material, table S2. ArcGIS Pro was used to prepare the data layers and to publish the layers to ArcGIS Online. The layers were then added to a web map, which was used within a simple web app, created using ArcGIS Experience builder, to view and explore the layers.

**Table 1.** Description of the different GIS layers used in this study and associated description.

layer	description activities/pressure levels	type of risk: source/receptor	references
UK disposal sites/aggregates site agreements	there are approximately 250 open dredged material disposal sites around the coast of England. Together, these sites receive many millions of tonnes of sediment per annum, mainly dredged from nearby ports, harbours and shipping channels. Although there are presently no data to support or refute this for the UK (reference cited are not UK-focused), it is possible that the dredged sediments disposed of to these sites contain elevated concentrations of microlitter as they are often located at the mouths of embayments or large estuaries.	source	[57,58]
wind farms	wind farms have been shown to be significant sources of microlitter to the marine environment from various processes (e.g. erosion processes).	source	[59–61]
oil and gas platforms	oil and gas infrastructures known to be a significant source for plastic materials (including microplastics) to the marine environment.	source	[62,63]
MPAs	microlitter detected in MPAs globally potential threat to protected marine ecosystems and biodiversity	receptor	[64]
spawning and nursery grounds	microlitter detected in fish globally. common sole *Solea solea selected as a model organism. Common sole* is a commercially important flatfish for which shallow inshore waters with fine sediments comprise important nursery habitats, and the diet of the juveniles is comprised primarily of infaunal invertebrates (e.g. polychaetes, bivalves, small crustaceans). Potential bioavailability of microlitter from sediment or via trophic transfer from contaminated infaunal invertebrates.	receptor	[12,13,65,66]
key benthic invertebrate species (OneBenthic species distribution model layers)	microlitter detected in benthic organisms globally. potentially higher bioavailability in higher accumulation zones for microplastics on the seabed. potential physical effects on benthic organisms following ingestion. potential trophic transfer of adsorbed co-contaminants (HOCs, metals, additives) to biota following ingestion.	receptor	[24,25,67–70]
	*Modiolus modiolus* (horse mussel): filter-feeder with long lifespan (decades), which risks ingesting suspended microlitter and accumulating adsorbed contaminants. May contribute to microlitter deposition through faeces production and sediment stabilization, particularly when at reef-forming densities. Its reefs are protected under national and international law. *Arctica islandica* (ocean quahog): very long-lived (400+years) filter- and deposit-feeder, potentially making it highly vulnerable to accumulated toxicity over time. Occurs at low abundance and does not form reefs, so is probably a minor contributor to microlitter deposition on the seabed. This species is legally protected and commercially harvested. *Sabellaria spinulosa* (Ross worm): reef-building filter-feeder that captures and traps microlitter within its biogenic structure, similar to *M. modiolus*. However, this species has a shorter lifespan (several years) for contaminant accumulation and can reach much higher densities, potentially leading to greater microlitter biodeposition. Its reefs are protected under national and international law. *Lagis koreni* (trumpet worm): deposit-feeder that can occur in dense numbers and consume large volumes of sediment, potentially including microlitter. A key food source for commercial fish, it may contribute to the transfer of microlitter and contaminants up the food web through to humans. No legal protections at present.		

HOCs: hydrophobic organic compounds

An investigation into the occurrence of microplastics in MPAs was also conducted by selecting four thresholds: 0–1000 (low), 1001–2000 (moderately elevated), 2001–3000 (elevated) and above 3001 (highly elevated) particles kg^−1^ d.w. sediment. Those thresholds were selected according to globally reported abundances of microplastics for the seabed as listed in table 2. Kukkola *et al*. [37] reported an abundance of microplastics as high as 2700 particles kg^−1^ d.w. sediment on the seabed for the UK. Additional information is given in electronic supplementary material, table S6.

**Table 2.** Reported abundance of microplastics in seafloor sediment for the North and Irish seas.

location	sampling year	sampling gear	sampling collection depth	number of samples (*n*)	abundance (number of particles kg^−1^ d.w. sediment)	analytical method	main polymer types	size (μm)	reference
southern North Sea	2014	Van-Veen grab	top 5 cm	46	2345.5 ± 254.3	ATR-FTIR and FPA FTIR imaging	PP, acrylates/polyurethane/varnish, and PA	20– 5000	Lorenz *et al.,* [71]
Dutch North Sea	2012– 2013	Van-Veen grab	top 10 cm	15	100–3600	micro-FTIR	NS		Leslie *et al*., [18]
North Sea and English Channel	2013–2014	Van-Veen grab	top 5 cm	27	0–3146	optical microscopy	NS	260 ± 194	Maes et al., [18]
southern England and Wales	2018	Corer	top 5 cm	8	215 ± 163 (seagrass) 221 ± 236 (unvegetated habitats)	optical microscopy	mainly fibres followed by films, fragments	NS	Unsworth *et al*., [72]
Danish part of the open North Sea	2015	Van-Veen grab	top 2 cm	10	192−675	20–5000	fibres (>90%), fragments	NS	Strand and Tairova, [73]
central North Sea, northern North Sea	2017	Van-Veen grab	top 1 cm	35	Central North Sea: 6800 ± 7600 (180 – 31 000) northern North Sea: 2500 ± 2900 (180–8800)	≥45	n.s	n.s.	Norwegian Environment Agency, [74]

PP: polypropylene, PA: polyamide, ** NS: not specified

## 3. Results and discussion

### (a) Mapping microplastics around the UK

The permutation importance plot for the full model is shown in electronic supplementary material, fig. S3. Thus, for example, salinity and wave velocity are the most important predictors in terms of permutation importance for predicting microplastic levels. The order of importance of variables from electronic supplementary material, fig. S3 was used to determine the best model. Starting with salinity, variables were successively added to the model and the LOOMAE calculated each time. For example, the third model contained salinity, wave velocity and silt. Electronic supplementary material, fig. S4 shows a plot of LOOMAE against number of variables. The minimum LOOMAE occurs when the model contains the five variables: salinity range (salinity), orbital velocity of waves at the seabed (wave velocity), percentage of fine sediment particles (silt), curvature of the seafloor terrain (Curv) and Nitrogen (< 2 mm) content (%m/m; Nit2). These variables were used to predict microplastic levels for the map (electronic supplementary material, table S3).

The partial plots (the effect of an explanatory variable with all the others held constant) for this model are shown in electronic supplementary material, fig. S5. The predicted abundance of microplastics is shown in (figure 2).

As highlighted, salinity, wave orbital velocity at the seabed, percentage fine sediment particles, the curvature of the seafloor terrain and nitrogen content were important variables in our model for the prediction microplastics distribution on the seabed (figure 2). Seafloor currents have been shown to play a crucial role in the transfer and storage of microplastics in the deep ocean [31]. Pohl *et al*. [75] investigated the importance of turbidity currents on the transport of microplastics. They concluded that turbidity currents tend to bury a high proportion of the microplastics they carry, and that seafloor settings at the termination of deep-sea submarine channels (i.e. channel, levee and lobe deposits and hadal trenches) may act as a sink that is highly concentrated in microplastics. The time scales over which this transport arises will depend upon the frequency of the turbidity currents that transit these systems [75]. Kane *et al*. [31] also indicated that thermohaline-driven currents, which build extensive seafloor sediment accumulations, can control the distribution of microplastics and create hotspots with the highest concentrations reported for any seafloor setting. They also reported generally higher abundance of microplastics where bottom currents occur [31].

### (b) Development of a risk-exposure map

**Figure 3.**
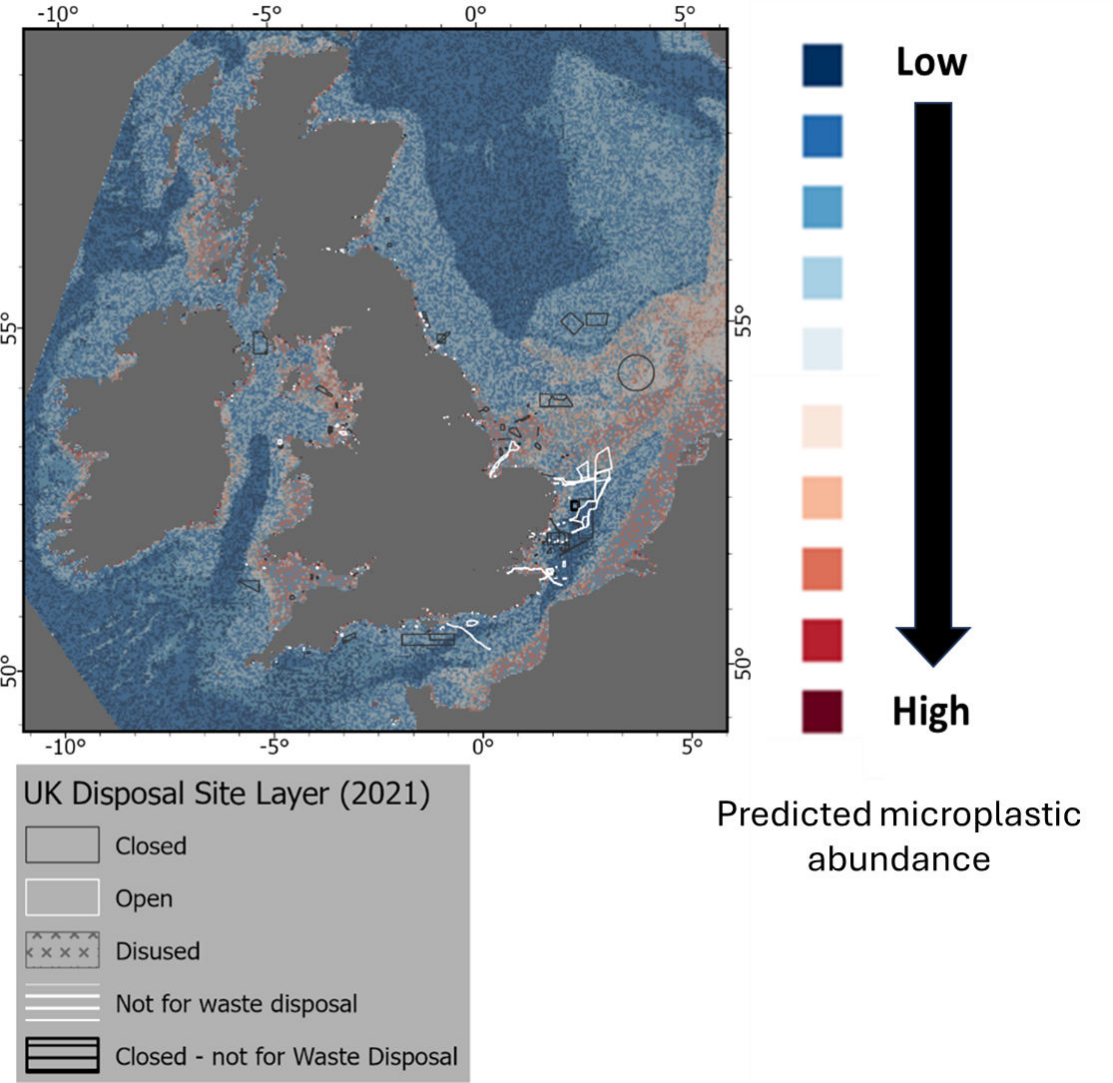
Location of disposal sites and predicted abundance of microplastics on the seafloor for the UK EEZ. GIS shapefile locations are available in electronic supplementary material, table S2.

**Figure 4.**
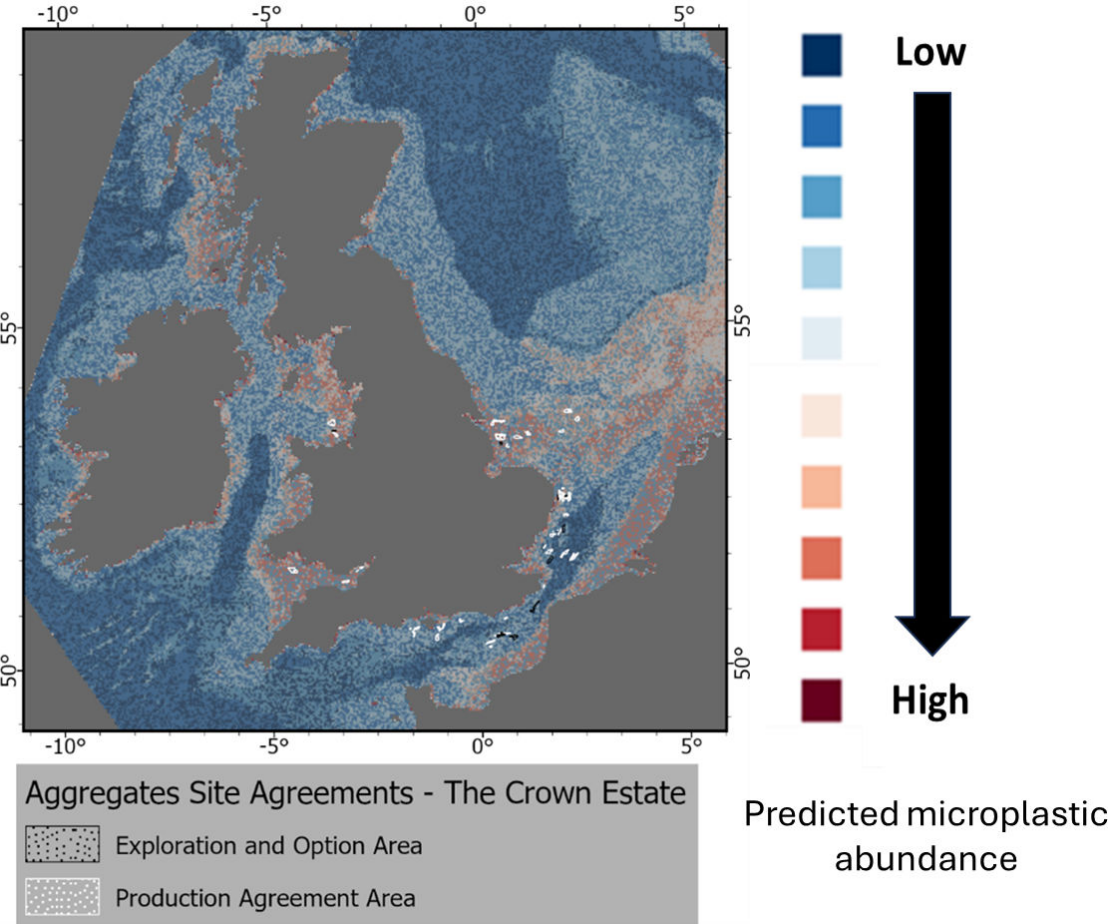
Location of aggregates site agreements for England and Wales and predicted abundance of microplastics on the seafloor for the UK EEZ. GIS shapefile locations are available in electronic supplementary material, table S2.

**Figure 5.**
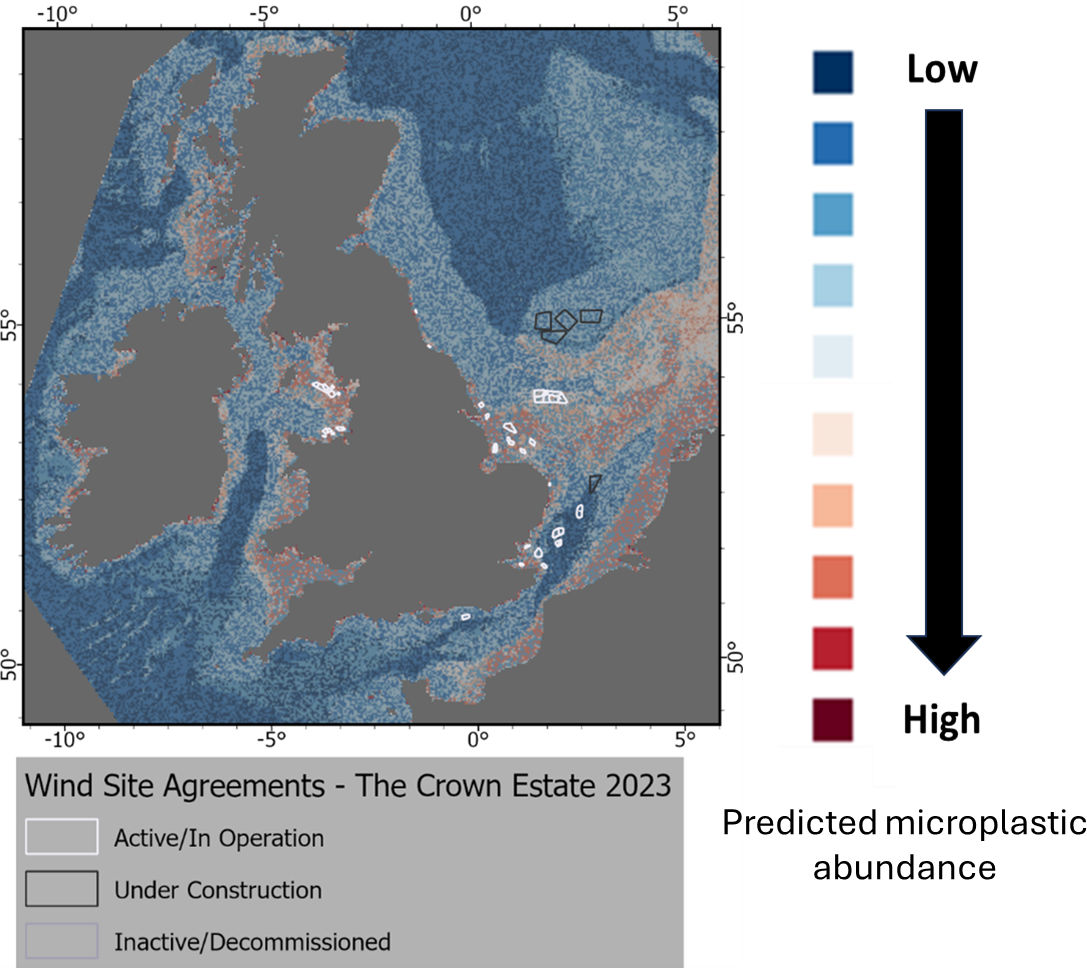
Location of wind site agreements for England and Wales and predicted abundance of microplastics on the seafloor for the UK EEZ. GIS shapefile locations are available in electronic supplementary material, table S2.

**Figure 6.**
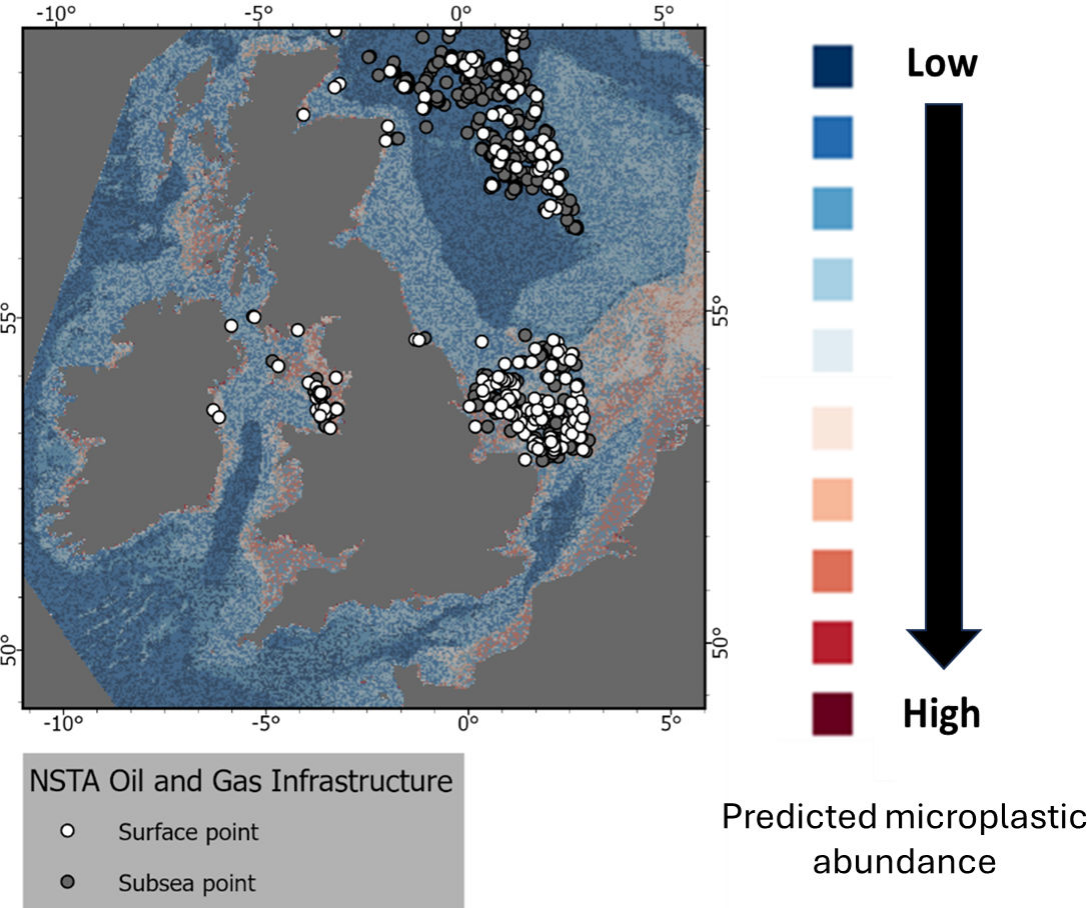
Location of North Sea Transition Authority (NSTA) Oil and Gas infrastructures and predicted abundance of microplastics on the seafloor for the UK EEZ.

**Figure 7.**
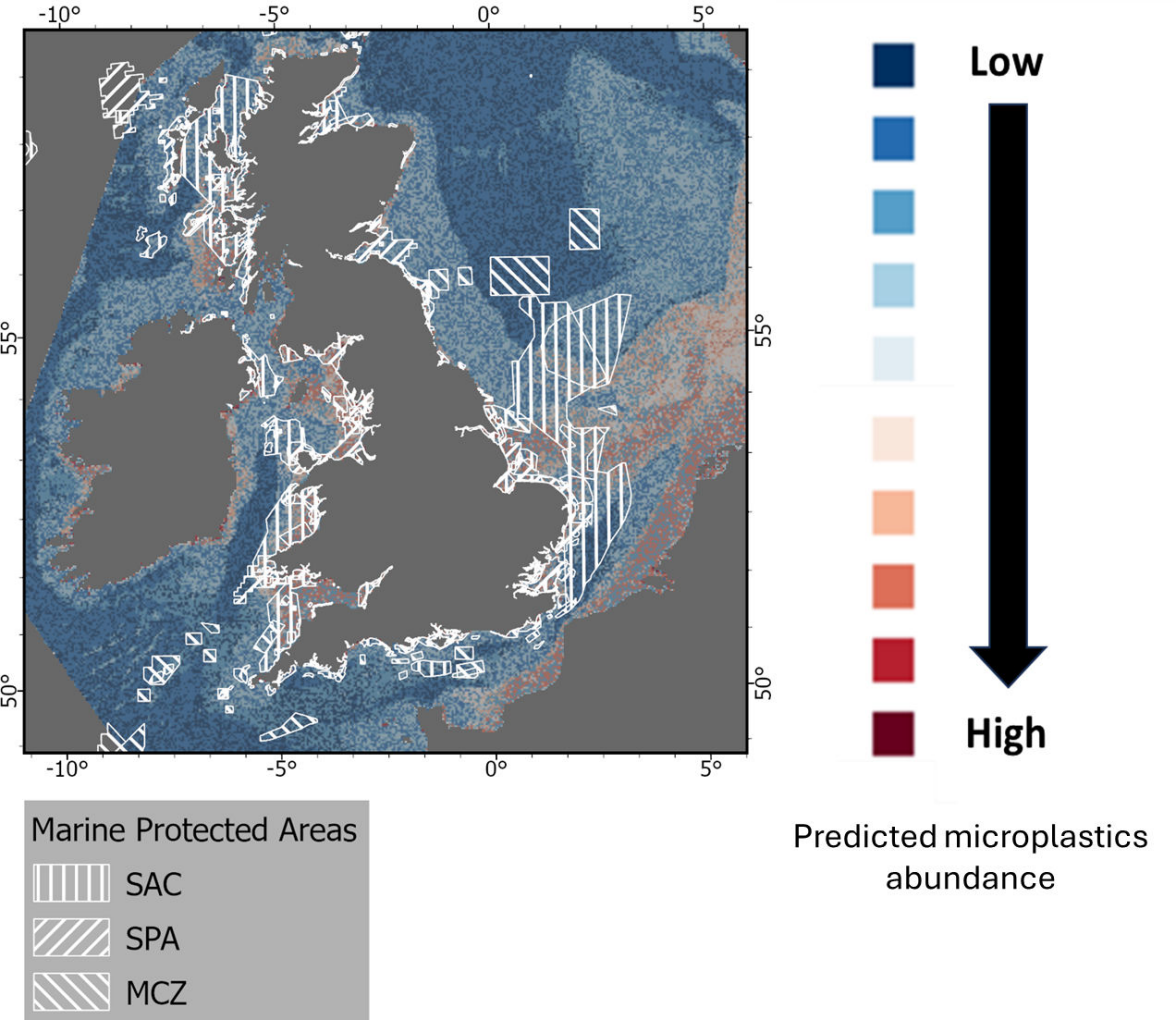
Location of MPAs for England and Wales and predicted abundance of microplastics on the seafloor for the UK EEZ.

**Figure 8.**
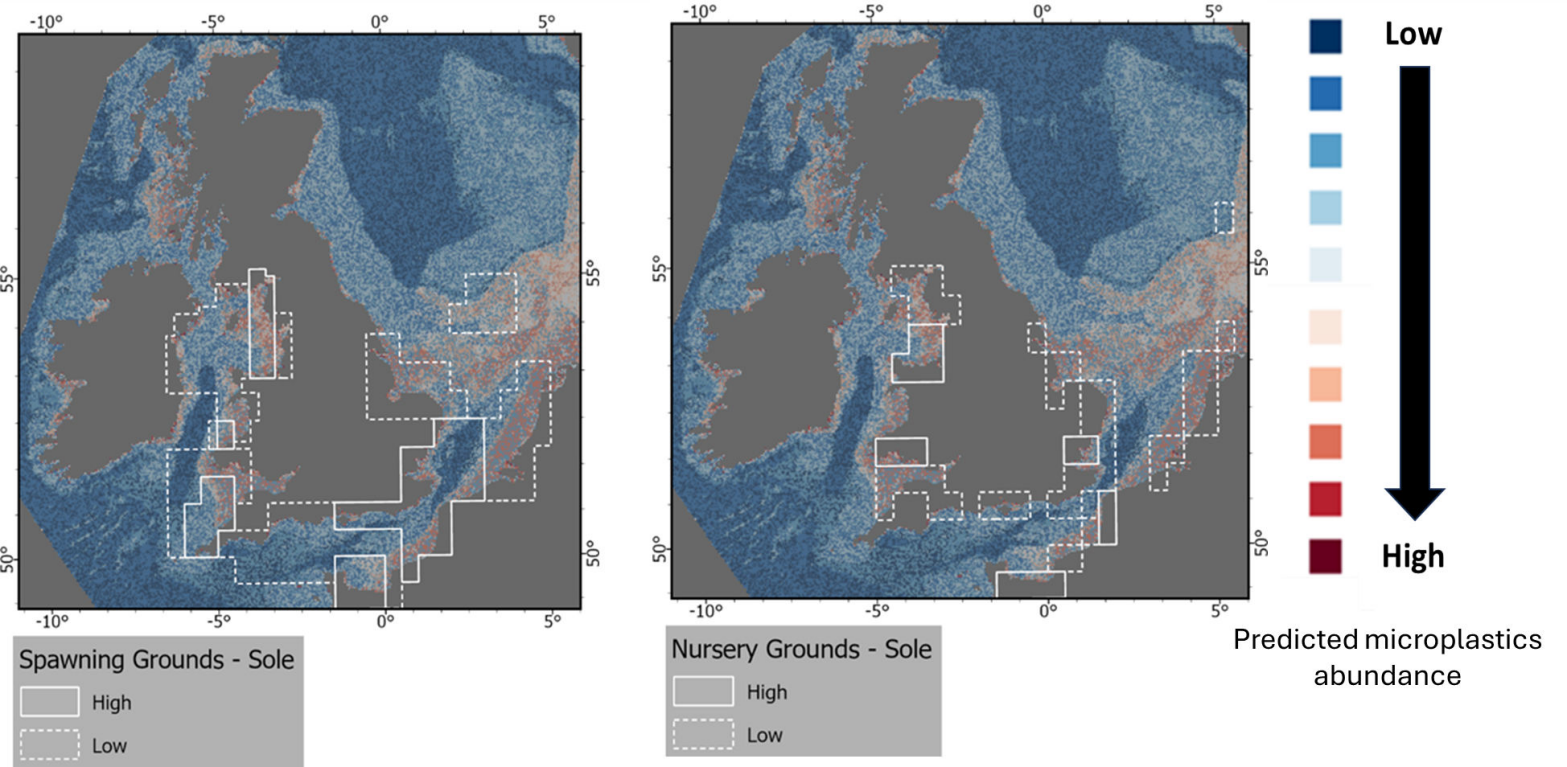
Spawning and nursery grounds for the Sole and predicted abundance of microplastics on the seafloor for the UK EEZ.

**Figure 9.**
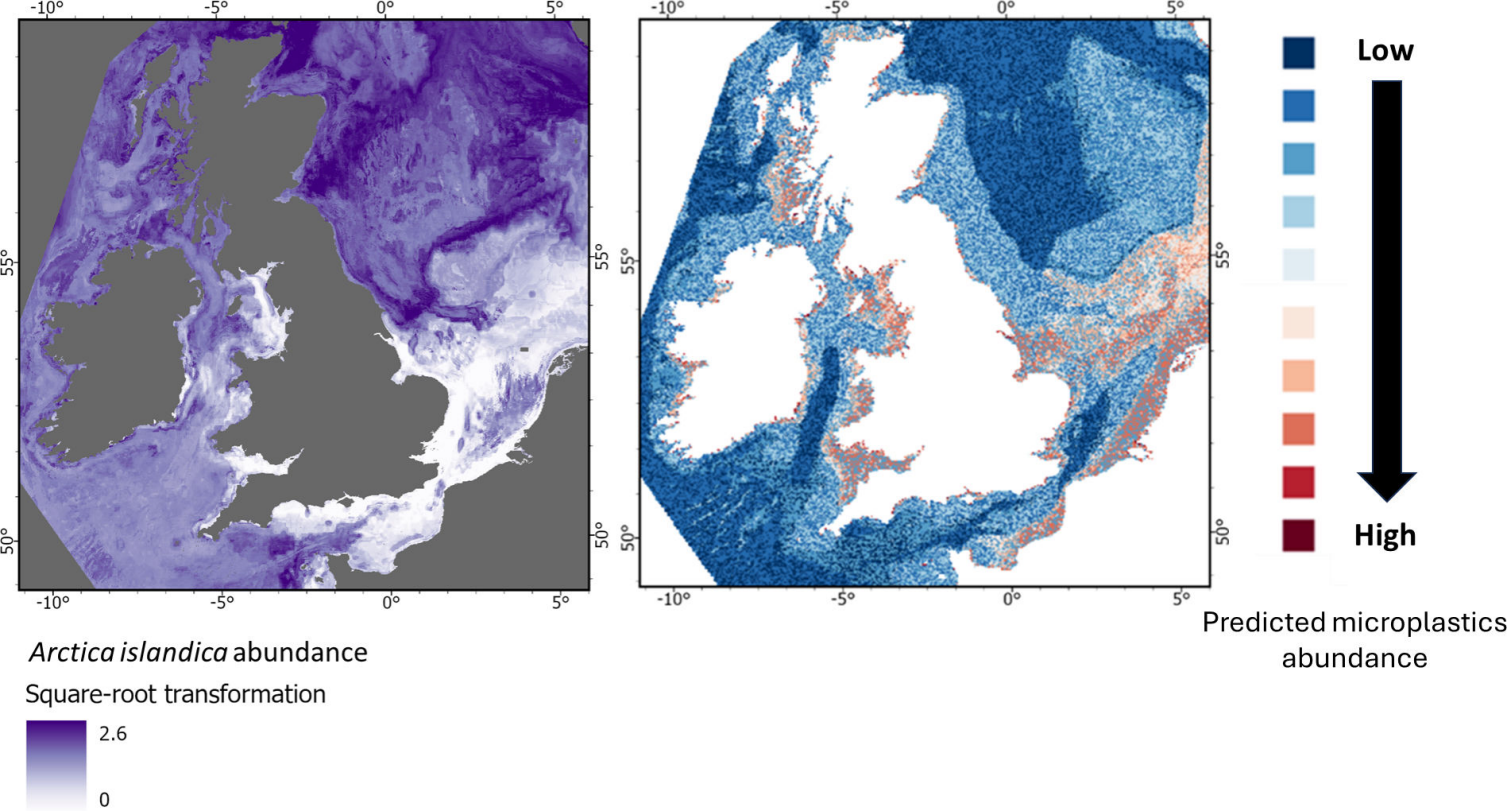
From top to bottom: Predicted abundance of *Arctica islandica*, *Modiolus modiolus*, *Sabellaria spinulosa* and *Lagis koren*i, shown alongside the predicted abundance of microplastics on the seabed for the UK EEZ.

Individual risk exposure maps are shown in figures 3–9 and were divided into ‘likely sources’ (e.g. disposal and dredging sites, marine infrastructures and associated processes) and ‘likely receptors’ (e.g. MPAs) as detailed in §3b,c.

### (c) Mapping microplastics and likely sources

The predicted abundance of microplastics for the UK ranged from 200 particles kg^−1^ d.w. sediment to approximately 3600 particles kg^−1^ d.w. sediment for the microplastics in the size range 20–5000 μm (figure 2). These microplastic concentrations were within the range of those previously reported for the north and central North Sea as well as the English Channel with concentrations of 0–3146 particles kg^−1^ d.w. sediment Meas *et al.* [18], 100–3600 particles kg^−1^ d.w. sediment [18] and 180–31 000 particles kg^−1^ d.w. sediment (Norwegian Environment Agency, 2018) (table 2). A recent study by Kukkola *et al*. [37] reported a mean concentration of microplastics (down to 10 µm) in sediment cores, collected in 2017. The authors reported concentrations as high as 1050 particles kg^−1^ d.w. sediment at the northwest of Jones Bank (Celtic Sea), 2700 particles kg^−1^ d.w. sediment at the Canyons (Celtic Sea) and 1190 particles kg^−1^ d.w. sediment at Dogger Bank (North Seas).

The identification of the likely sources of microlitter is complex due to their relatively small sizes, their wide range of sources as well as their mobility in the marine environment [76]. Previous work focused on the identification of accumulation zones for microlitter contamination (in the size range 20–5000 μm) for the UK (England and Wales). Consistently significantly (*p* < 0.05) higher concentrations of microplastics were observed near to rivers and harbours, especially for CSEMP stations 245 (Off Tyne), 345 (Humber/Wash), 376 (Off Tyne), 655 (Cardigan Bay), 536 (Lyme Bay), 575 (Off Tamar) and 715 (Liverpool Bay) [11]. Additional information on CSEMP stations is provided in electronic supplementary material, fig. S1 and table S1. An earlier study from Green & Johnson [77] reported the occurrence of microplastics (≥ 100 μm) in all sediment samples collected from England’s inshore waters as well as in 61% of samples in MPAs. The authors reported the highest frequency of occurrence of microplastic particles in sediment samples collected from Runswick Bay Marine Conservation Zone (MCZ) located on the north-east coast of England, and from Upper Fowey and Pont Pill MCZ (located on the south-west coast of England), where 93.3% and 92.1%, respectively, of the samples collected contained plastic. The highest mean density of microplastic particles was observed in the Mersey Estuary SPA, where there was an average of 42.67 particles kg^−1^ d.w. sediment 0.1 m^−2^ [77]. High densities of microplastics were also reported at remote sites, as well as for those closer to urban or industrialized areas [77]. Observations from Green & Johnson [77] were in agreement with previous observations from Bakir *et al*. [11] for similar sampling areas, with a high occurrence of microplastics for CSEMP stations 245 (Off Tyne), 536 (Lyme Bay) and 715 (Liverpool Bay).

The investigation of zones potentially at risk was carried out by overlaying the predicted distribution of microplastics layer with some specific layers (e.g. UK disposal site layer, wind site agreements) as detailed in electronic supplementary material, table S2. The types of risks were defined as either ‘source’ (i.e. activities contributing to an increase in abundance of microlitter on the seabed) or ‘receptor’ (i.e. zones at potential risk from an elevated abundance of microlitter on the seabed such as benthic organisms or MPAs). Note that in the present study it is not possible to quantify the relative contribution of the different sources to an increased abundance of microlitter on the seabed.

#### (i) Disposal and aggregates sites

Locations of the UK disposal sites as well as aggregates sites agreements are shown in figures 3 and 4. Little is known about the occurrence and fate of marine plastic litter, including microplastics in dredge materials due to the scarce scientific evidence currently available [78,79]. A case study in the Thames and Medway estuaries, UK, highlighted a decrease of the presence of microplastics seaward, with a strong correlation with organic carbon (%) but not particle size. In the Thames, there was a clear link in accumulation related to distance from urbanization as well as site dynamics [80]. The monitoring of microplastics in port sediments in France under REPOM, a national port sediment quality monitoring network, was presented at the SedNet 2023 conference with an evaluation of methods being used for microplastic analysis [81]. There is a need for additional monitoring data for the understanding of the environmental effects for these plastic materials for risk assessment purposes [82]. Sediments are known to be the final sink for microplastics, and high concentrations are expected to occur for sediments in harbours, ports and navigation channels due to localized important sources of litter (anthropogenic activities, wastewater treatment plants, accidental spillages and littering). Furthermore, little is known on the contribution of dredging activities on the resuspension of macro and microplastics to the coastal and marine environment with potential bioavailability to marine biota with unknown effects. Data on concentrations are necessary for dose–response effects for marine organisms and ecosystems to estimate ‘safe’ environmental concentrations. It is possible that due to the relatively low density of microplastics compared with sediment, much of the microplastic will resuspend in the water phase, and be deposited back on to the site of dredging, rather than at the disposal site. However, different sediment types (e.g. gravel, sand or silt/clay) will retain plastic particles with different efficiency with silt/clay matrices acting as ‘traps’ for these items and will be transported accordingly. The lack of a risk assessment for microplastics in dredged materials is making prioritization of policy drivers difficult. There is, however, enough information to suggest that microplastics can cause a physical and chemical effect on marine organisms following direct exposure or ingestion [24,25,27,83]. Some scenarios can be proposed for the reduction and/or removal of macro and microplastic litter from dredged materials and include reducing/removing sources and entry or marine plastic litter and by the removal of macrolitter. The removal of macrolitter could be achieved prior to waste transport to the disposal site or before dredging operations take place using divers or bottom-scraping devices such as trawl nets or hydraulic dredges [82]. Separation of large and small plastic materials from dredged materials can also be achieved using excavator buckets or fixed screens.

#### (ii) Marine infrastructures (wind farms)

Marine infrastructures have been suggested as a potential source of microlitter in the marine environment [67,84–86]. The erosion of wind turbine blades in particular, has been characterized as a potential sources of plastic pollution with the production of 30 to 540 g per year per blade [85]. The installation and presence of wind farms could also disturb the stability of the seabed by increasing the seabed shear stress and in turn increasing the mobility of microplastics associated with the sediment [86]. Plastic components on offshore infrastructure are varied and have been previously categorized as thermal insulating materials (e.g. polyurethane foam), electrical insulating materials (e.g. polyvinyl chloride (PVC)), protection against corrosion (e.g. fusion bonded black polyethylene (FBBP), high build epoxy (HBE) used in paints and coatings and filling materials (e.g. polyamide, HDPE) [84]).

The UK wind site agreement layer was overlaid with the microplastic layer as shown in figure 5. Wind site agreements were divided between active/in operation, under construction and inactive/decommissioned. It is, however, not possible, based on visual observation alone, to conclude whether the presence of wind farms for a specific location does contribute to a higher abundance of microlitter on the seabed.

Generally, a higher predicted abundance of microplastics was identified for locations with operational wind farms (figure 5). In the Irish Sea, a higher predicted abundance of seafloor microplastics corresponded to the Barrow Offshore wind Farm (fully commissioned in July 2006), West of Duddon Sands wind farm (October 2014) as well as the Rhyl Flats Offshore Wind farm constructed within Liverpool Bay (December 2009).

While most active wind farms corresponded to areas with a higher abundance of seafloor microplastics, a number of locations corresponded to a lower abundance of microplastics, for example East Anglia One (off the Suffolk coast and operational from July 2020). However, we note that a relatively high abundance of floating micro, meso and macroplastics were recorded, suggesting an accumulation zone for plastic items for that area (off the coast of Lowestoft— East of England) [19]. For the North Sea, Hornsea 1 was fully commissioned in December 2019. The Hornsea 2 wind farm began operation in August 2022. Assuming Hornsea 1 is already contributing to the microplastic levels in the area, then we would theorize that Hornsea 2 would increase these levels further. The wind farms Dogger Bank A, Dogger Bank B and Sophia, all currently under construction at the time of writing, are located between 130 km and 190 km from the north-east coast of England at their nearest points [59]. Farther south the East Anglia Three wind farm, located approximately 69 km from the Suffolk coast [60], is due to begin construction. A lower abundance of microplastics was predicted for these areas (figure 5). Additional sampling in these areas would allow comparisons to be made helping to investigate any increase in abundance due to the construction process (e.g. disturbance of the seabed or degradation processes from the marine infrastructures).

Any microplastics emitted by wind farms and other offshore structures may be deposited or accumulate a considerable distance from the original site. Assuming that the greatest erosion of turbine blades takes place during operation we could expect particles to be transported many kilometres from the turbine due to the strong winds. Local physical and oceanographic processes (i.e. wind speed and currents) alongside plastic physical properties (e.g. size, morphology, density etc.) will also directly influence the short- to long-term transport of floating particles for the area and promote surface dispersion rather than vertical settling and deposition on to sediments.

#### (iii) Marine infrastructures (oil and gas platforms)

Locations of the North Sea Transition Authority (NSTA) oil and gas infrastructures are shown in figure 6 with a higher density of platforms located in the southern North Sea and Irish sea. Generally, a higher predicted abundance of microplastics on the seabed coincided with a higher density of oil and gas infrastructures. It is, however, not possible to correlate or quantify the contribution of such activities to the presence of microplastics on the seabed without any targeted monitoring programme. Offshore oil and gas infrastructures have been identified as important sources of plastics and microplastics to the marine environment with some related activities potentially contributing to the increase of microplastics on the seabed [61,87]. OSPAR identified the OSPAR regions North Sea and Celtic Seas to be under important pressures from oil and gas activities [88]. Furthermore, the presence of offshore oil and gas infrastructures alters currents locally causing a higher percentage of silts to build up in the current ‘shadow’ of structures [89]. These changes in settling velocities for sediment particles will have implications for microplastic settlement although this is both complex and under-researched as it will probably be a combination of numerous biotic and abiotic factors [62].

Offshore oil and gas infrastructures are covered in paints and coatings that contain plastic. These coatings are added prior to deployment and last the entire lifespan of the development until the cutting or disruption of the structure offshore for repair or decommissioning. Coating and paint materials are not part of the OSPAR Oil and Gas Chemicals remit [63,90]; however, other chemicals containing plastics are used in the drilling and extraction processes, some of these are discharged under license and therefore technically are not considered litter but are discharged throughout the lifetime of a project [91]. Plastics are useful and flexible and can be used as fluid loss control materials (LCM), they are deployed to plug cracks and fissures in the formation and improve the flow of hydrocarbons and efficiency of the well. LCMs containing plastics may be used in water or oil-based drilling muds but they cannot be discharged to the marine environment. The banning discharge of plastic containing LCM was agreed at OSPAR’s Offshore Industry Committee meeting in 2013.

Other plastic containing offshore chemicals are still discharged, the flexibility of plastics are useful in cementing to prevent hydrocarbon migration, which is particularly important in decommissioning and repurposing for carbon capture storage applications. Cementing in the offshore oil and gas sector allows a maximum of 10% cement mix water discharge, but up to 100% for poor quality mixtures; these fluids often contain plastics along with the solids needed for cementing [92].

During the life of hydrocarbon installations, well stimulation may also be carried out, often by fracturing. Proppants are often plastic resin coated to allow the proppant particles to slide over each other, while these are designed to remain in the formation and hold open the cracks some are discharged through formation losses and back pressure, up to 40% of the proppants could be discharged with their plastic coatings, some of these will be captured and recycled for other fracturing operations or shipped to shore for disposal [93]. Plastics and microplastics are also used as flow improvers and other chemicals to reduce drag and break emulsions, most of these chemicals are highly hydrophobic and are unlikely to be discharged with the chemicals designed to follow the hydrocarbon stream rather than be discharged in produced water. Either by design or due to general chemical properties, most plastic and microplastics are not discharged to the marine environment, thereby reducing plastic and microplastic effects. Where there are discharges these are controlled with a view to minimize plastic effects at the point of issuance of permits, these controls are implemented through the OSPAR agreements such as the OSPAR Harmonised Mandatory Control System and national measures to reduce pollution.

### (d) Mapping microplastics and likely receptors

Seabed habitats provide valuable ecosystems which experience increased anthropogenic pressures from a number of activities often leading to cumulative effects [94]. Pressures and activities on the seabed can be accumulative and varied from smothering (e.g. disposal of dredged materials, cuttings from oil and gas exploration), obstruction (sealing) (e.g. oil and gas platforms, wind turbines, wrecks, telecommunication and power cables), abrasion (e.g. benthic fishing using trawl gear) to extraction (e.g. aggregate extraction) [95]. Addressing the risk of microplastics to the marine environment is difficult due to the lack of risk assessment frameworks taking into account the complexity of microplastics in the environment (e.g. size, morphology, polymer type, densities and chemical composition) as well as comparison with natural particles [96].

#### (i) Occurrence of microlitter in marine protected areas (MPAs)

MPAs are areas of the ocean established to protect habitats, species and processes essential for healthy, functioning marine ecosystems. In England, MPAs are designated to protect marine ecosystems or specific habitats or species (also known as ‘features’) and have conservation objectives which state which conservation outcomes the MPA is designed to achieve. There are presently 178 MPAs in English waters, covering 51% of inshore and 37% of offshore waters, including Special Areas of Conservation (SACs), Special Protection Areas (SPAs) and Marine Conservation Zones (MCZs) [97,98].

The predicted distribution of microplastics in our model indicated the occurrence of high concentrations within MPAs, especially for the West Wales Marine (SAC), Outer Thames Estuary (SPA), Greater Wash (SPA), Bristol Channel Approaches (SAC), Liverpool Bay (SPA), West of Copeland (MCZ), West of Walney (MCZ) as well as some parts of the southern North Sea (SAC) and Holderness Offshore (MCZ) (figure 7). A lower abundance of microplastics was also predicted for the Dogger Bank (SPA). No data were collected/available for the other MPAs.

Green *et al*. [77] also reported the occurrence of microplastics in 61.2% of sediment samples collected in MPAs. They reported the highest densities of microplastic particles in intertidal estuarine MPAs such as Upper Fowey and Pont Pill MCZ, Exe Estuary SPA and Mersey Estuary SPA. For this study, an investigation into the abundance of the occurrence of microplastics indicated that 39% of MPAs had a ‘low’ mean abundance of microplastics (in the range 0–1000 particles kg^−1^ d.w. sediment), 58% in ‘moderately elevated’ areas (1001–2000), 4% in ‘elevated areas’ (2001–3000) and 0% for ‘highly elevated’ areas (above 3001 particles kg^−1^ d.w. sediment) (electronic supplementary material, table S6). Further observations are, however, needed for a more in-depth analysis of the occurrence and abundance of microplastics in MPAs which is planned under future work. Microplastics have also been reported in MPAs globally highlighting their ubiquity in protected areas [99,100]. The widespread distribution of microplastics in MPAs is perhaps not surprising as they were not designated for reducing marine litter and microplastics; however, it raises an important question as to how we best protect sensitive habitats from this pervasive pollutant.

#### (ii) Risk exposure of microlitter for fish

While the ingestion of plastics by fish has been long known [101], there has been increased interest in recent years [102]. A range of factors may influence the quantity and types of microplastic ingested, including fish size, mouth size and gape, trophic guild and feeding strategy, and habitat use [102].

Modelled outputs indicated that microplastics would be more prevalent in inshore waters, including sites such as the Bristol Channel, eastern Irish Sea and Outer Thames (figure 8). Such areas are known to form important nursery areas for a range of fish species, including a range of demersal elasmobranchs, and demersal and small pelagic teleosts [64]. High densities of microlitter in sediments could, therefore, result in the accidental ingestion of microplastics by those species of fish that are associated with the seafloor, especially those that feed on infaunal prey, or that may ingest sediments when feeding, or when burying in surficial sediments.

Common sole *Solea solea,* is a commercially important flatfish for which shallow inshore waters with fine sediments comprise important nursery habitats, and the diet of the juveniles is comprised primarily of infaunal invertebrates (e.g. polychaetes, bivalves, small crustaceans) [103]. Indeed, studies in the Adriatic Sea have indicated a high prevalence of microplastics in sole (95%; *n* = 533 [104]).

European flounder *Platichthys flesus,* is a coastal and estuarine species, and studies in the Thames have reported that 84.8% (*n* = 66) of specimens examined had ingested plastic, ranging from 71−90% of individuals sampled in different samples [105]. Horton *et al*. [15] reported a lower proportion (37.5%) from the Rivers Thames and Stour, but this was based on a smaller sample size (*n* = 16).

These high occurrences of plastic ingestion in estuarine waters is in contrast to the lower proportions observed for fish larvae however (*n* = 347, 2.9%, ranging from 0.7−5.3% for three sampling sites [106]), and fish (*n* = 504, 36.5%, ranging from 23.5−51.9% for the 10 species analysed [107]) sampled off Plymouth (western English Channel). Consequently, those fish species that utilize coastal and transitional waters may be at greater risk of exposure to microplastics. In addition to this potentially affecting the juveniles of marine fish on coastal nursery grounds, it will also probably effect on those fish species that are more estuarine dependent, such as the grey mullet (Mugilidae). The incidences of plastic ingestion for members of this family elsewhere in the world have been reported in the range of 57−75% [65,108].

Future studies could usefully examine inter-specific differences in the proportion of individual fish containing microplastics for case-study sites and identify which fish species (and length categories) or trait categories, may be more suitable as possible indicators for any future monitoring of at risk species. Furthermore, an improved understanding of the longer-term implications of microplastics on fish populations is required.

#### (iii) Risk exposure of microlitter for benthic invertebrate species

**Figure 10.**
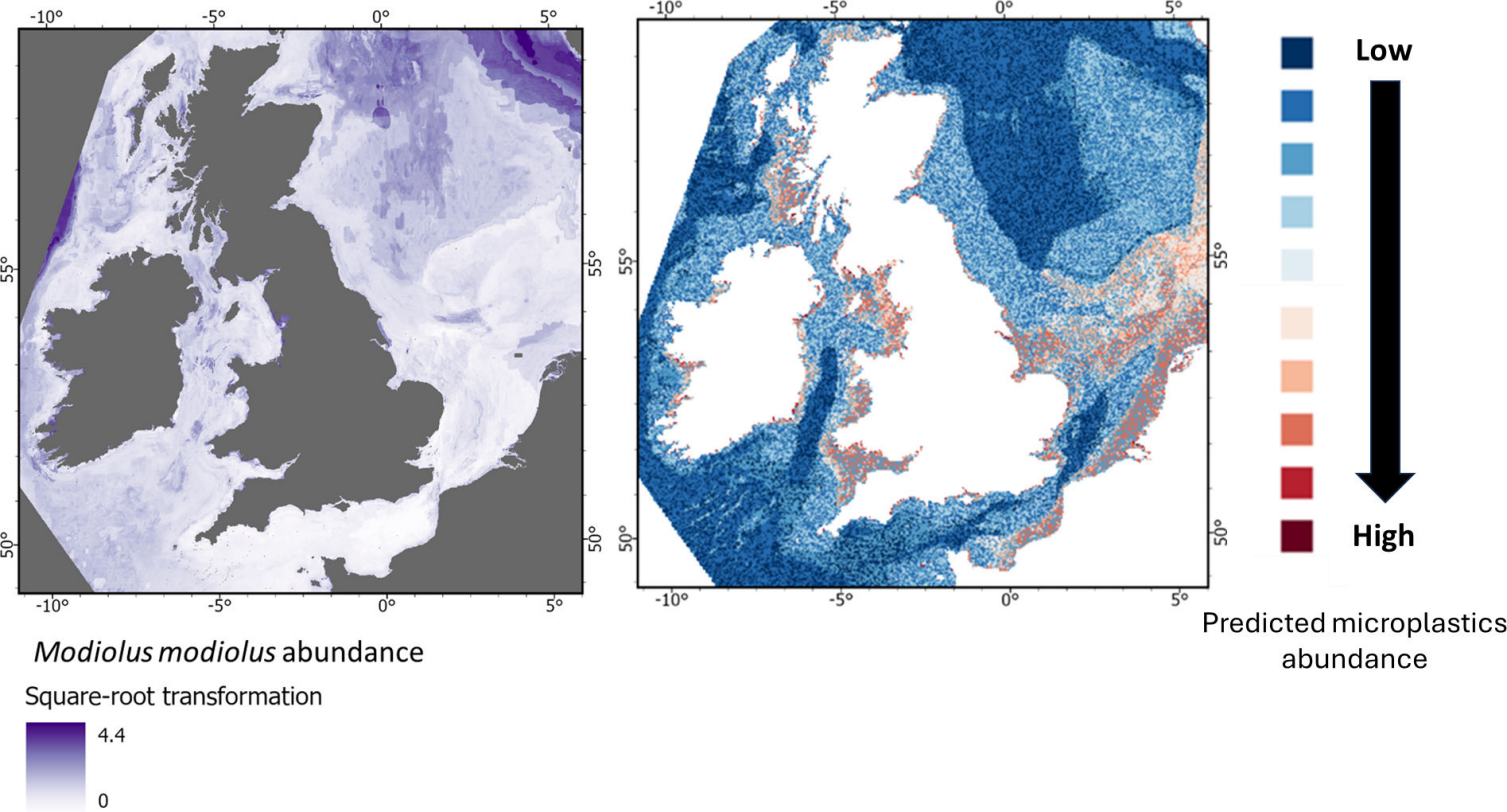
From top to bottom: Predicted abundance of *Arctica islandica*, *Modiolus modiolus*, *Sabellaria spinulosa* and *Lagis koreni*, shown alongside the predicted abundance of microplastics on the seabed for the UK EEZ.

**Figure 11.**
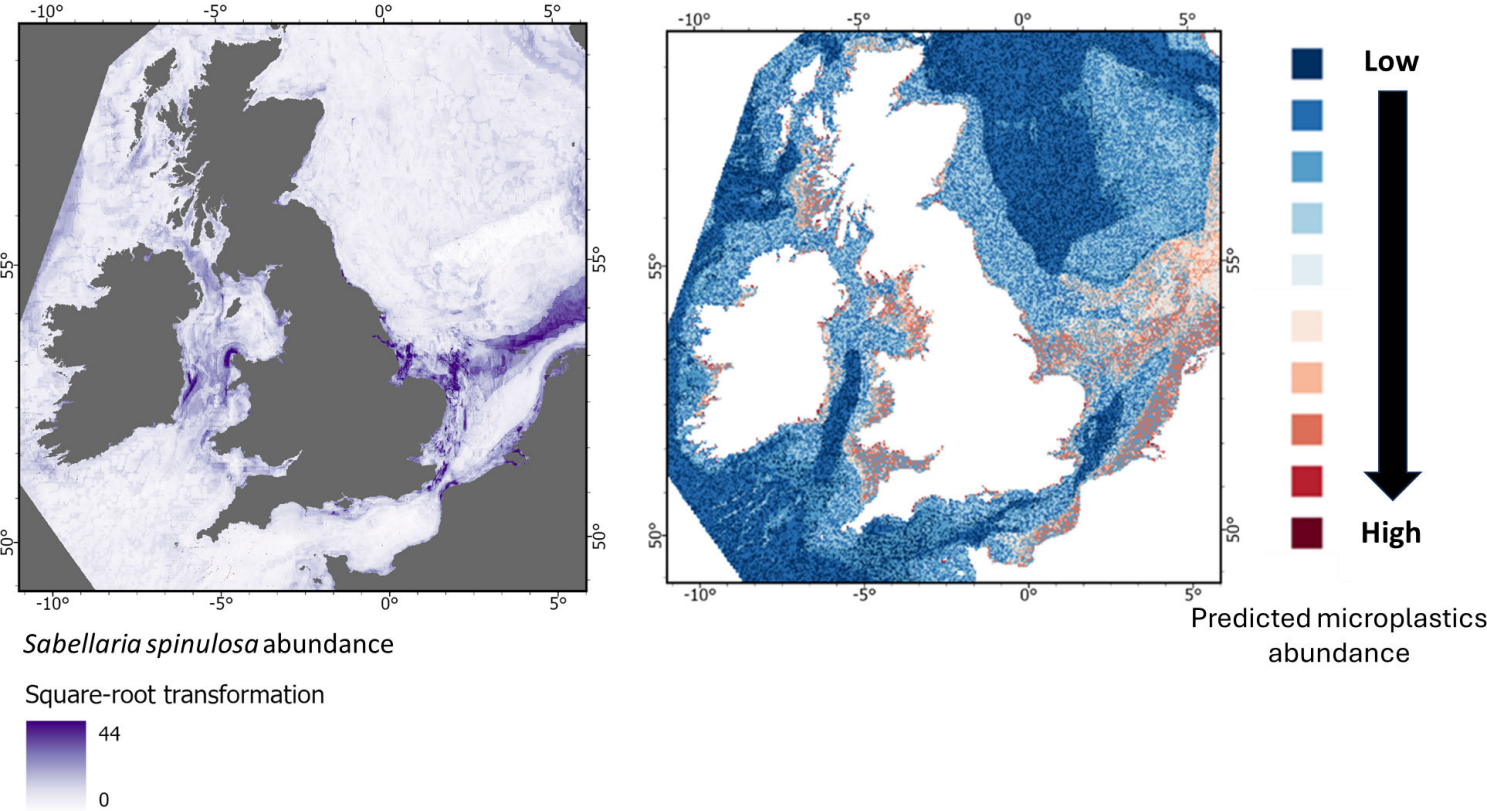
From top to bottom: Predicted abundance of *Arctica islandica*, *Modiolus modiolus*, *Sabellaria spinulosa* and *Lagis koreni*, shown alongside the predicted abundance of microplastics on the seabed for the UK EEZ.

**Figure 12.**
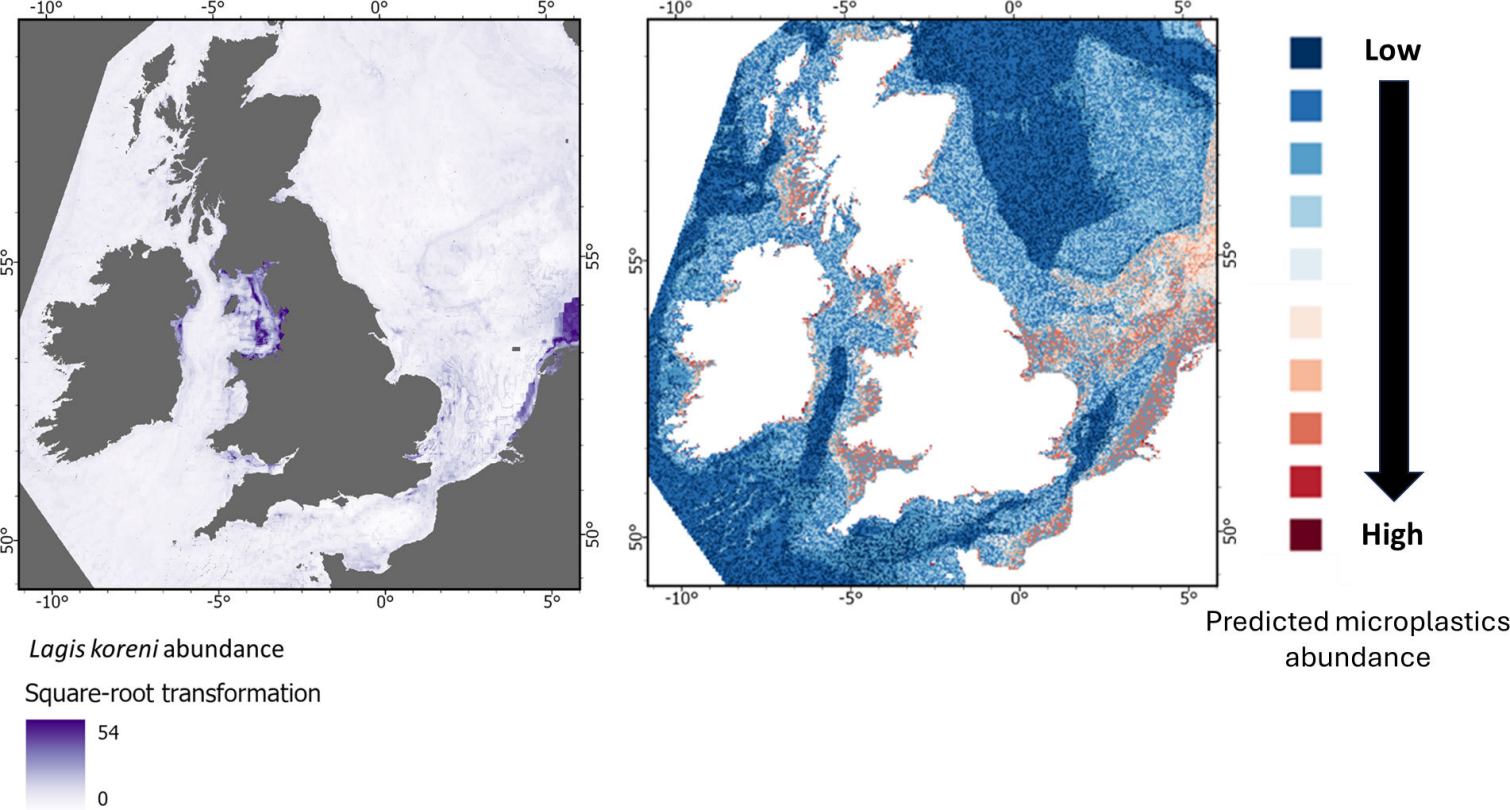
From top to bottom: Predicted abundance of *Arctica islandica*, *Modiolus modiolus*, *Sabellaria spinulosa* and *Lagis koreni*, shown alongside the predicted abundance of microplastics on the seabed for the UK EEZ.

The modelled distribution of microlitter implies differing levels of risk for the four key benthic invertebrate species considered (figures 9–12). The bivalve *Arctica islandica* (ocean quahog) is largely restricted to offshore waters and is absent from nearshore areas where predicted microlitter levels are typically high, suggesting minimal direct exposure (figure 9). Another bivalve, *Modiolus modiolus* (horse mussel), is most prevalent in the northeast of the study area, where microlitter concentrations are relatively low (figure 10). However, less expansive populations of this species are projected in nearshore areas with elevated microlitter levels, indicating potential localized effects despite limited overall risk.

By contrast, the polychaetes *Sabellaria spinulosa* (ross worm) and *Lagis koreni* (trumpet worm) appear to be far more exposed (figures 11 and 12). High densities of *S. spinulosa* are projected in nearshore waters and an offshore band of the southern North Sea with elevated microlitter levels (figure 11). Similarly, *L. koreni* is projected to be most abundant in nearshore areas with substantial microlitter contamination (figure 12). Given that *S. spinulosa* can trap microlitter and adsorbed contaminants within the seabed and *L. koreni* may facilitate its transfer up the food web, their interactions with litter could potentially have broader ecological consequences. The risks posed may therefore extend beyond these species to the associated biodiversity and ecosystem services they support.

Research on microlitter interactions with benthic invertebrates has predominantly focused on intertidal or shallow subtidal species, such as *Arenicola marina* (lugworm) and *Mytilus edulis* (blue mussel) [109–111]. While these species inhabit different environments than the deeper-water species considered here, their functional similarities imply potential parallels in microlitter sensitivity. Feeding mode has been shown to be a significant predictor of microplastic uptake by benthic marine invertebrates globally with omnivores, predators and deposit feeders exhibiting the highest body burdens [68] demonstrating that biological traits can be used to explain microplastic body burdens across species and spatial scales. For instance, *M. edulis* is closely related to *M. modiolus* and exhibits comparable particle uptake mechanisms, while *A. marina* shares a similar feeding strategy with *L. koreni*, though it primarily ingests particles from deeper within the sediment. However, direct evidence of microplastic ingestion and its biological effects in these deeper-water species remains limited. Targeted monitoring surveys aimed at quantifying these interactions, particularly in areas where species and or/ trait abundances coincide with high microlitter concentrations, would help confirm species–specific risks and shed light on any broader ecological consequences.

## 4. Summary and conclusion

This study presents a novel approach for identifying zones at ‘risk’ from the accumulation of microplastics and proposes the basis upon which to prioritize sites for both monitoring and for direct management measures. It is evident from our findings, together with those from previous studies [37,77] that microplastics are widespread and abundant on the seabed within the United Kingdom Exclusive Economic Zone (UK EEZ). This is also true to a large suite of MPAs currently designated to protect specific habitats or species of conservation interest. While this study only considered individual sources, it is clear their cumulative effects will greatly influence the formation, transport and distribution of microplastics on the seabed [95].

To improve our capacity to build more robust maps of microlitter in marine sediments for the UK, there is an urgent need to increase the evidence base of the abundance of microplastics within the seabed. The acquisition of marine sediment microplastic data only started as recently as 2017, and currently, direct empirical data are only available for approximately 50 sites. However, this evidence base is continually expanding through the collection of new samples each year. Incorporating these data, as they become available, will result in improved maps of microplastic concentrations, with greater spatial resolution, in the future. Importantly, gap filling of spatial areas, depths and hydrographic settings that are under-sampled will be critical for understanding the depth horizon at which microplastics tend to be distributed. Having systematic measures of the parameters that have been found to be good microplastic storage potential descriptors will also be important to improve further modelling approaches and can be used to target sampling areas to improve model accuracy. It is important to note an inherent limitation of the present study as it only considers microplastic storage potential: it does not include source of microplastic, category, shape or other important features and how these vary through space and time. Considering the sources and the variation in the absolute quantities will allow actual microplastics abundance to be quantified within the seabed. Future work may also include additional maritime activities of interest and potential stressors in addition to microlitter to enable the investigation of combined effects rather than the isolated assessment of individual layers. Other biotic parameters, not included in the present study, have also been reported to influence the transport and fate of microplastics in the marine environment and influence their settling velocity to the seabed. Interaction of plastic particles with biota as well as with suspended organic matter will also affect the density of particles and affect their settling velocity and association with seafloor sediment, and will be incorporated in future work [69,112,113].

## Data Availability

Data are available at [114] and [115]. Supplementary material is available online [116].
